# Editorial: Vestibular Migraine

**DOI:** 10.3389/fneur.2017.00213

**Published:** 2017-05-19

**Authors:** Mark Obermann

**Affiliations:** ^1^University Hospital Essen, University of Duisburg-Essen, Essen, Germany

**Keywords:** vertigo, vestibular migraine, headache, neuro-otology, dizziness

**Editorial on the Research Topic**

**Vestibular Migraine**

Vestibular migraine (VM) is now the accepted name for vestibular symptoms that are causally related to migraine. The International Headache Society and the International Bárány Society for Neurootology together have developed a consensus document with diagnostic criteria for VM (1), but the existence and the relation to migraine is still disputed by some clinicians and researchers alike. Our research topic VM tried to shed more light on the clinical features as well as pathophysiological origin of this interesting and unique disorder. Different aspects of this disorder were highlighted and included genetics, pathophysiology, clinical phenotype in adults and children, diagnostic findings, as well as treatment options. There are solid indications that VM shares at least some pathophysiological features with migraine, but also entails some unique features that separate it from migraine with or without aura and underline its claim as specific disease entity.

For those that acknowledge the existence, VM is considered the most common cause of recurrent spontaneous vertigo attacks. It has a lifetime prevalence of about 1% and a one-year prevalence of 0.9% in the general population (2) and accounts for about 7% of patients seen in dizziness clinics and 9% of patients seen in migraine clinics (3). Nevertheless, it is still underdiagnosed and not well understood. It has been proposed that VM has a genetic cause, namely an autosomal dominant pattern of inheritance with decreased penetrance in men (4), but a lot of research is needed to further clarify its underlying pathophysiology and the relation to migraine in this regard. Clinical studies on specific diagnostic clues and therapeutic agents specific for the treatment of VM only just started and will have to be intensified in the future.

## Author Contributions

The author confirms being the sole contributor of this work and approved it for publication.

## Conflict of Interest Statement

The author declares that the research was conducted in the absence of any commercial or financial relationships that could be construed as a potential conflict of interest.
